# Complete genome sequence of psychrobacter sp. KFRI-CH2-11: A psychrotolerant bacterium with probiotic, biofortification, and antimicrobial potential for the dairy and meat industries

**DOI:** 10.1016/j.dib.2025.111344

**Published:** 2025-01-28

**Authors:** Myunglip Lee, Yucheol Kim, Hae-Won Lee, Yukyoung Park, Sunghun Yi

**Affiliations:** a Nongsaengmyeong-ro, Iseo-myeon, Korea Food Research Institute (KFRI), Wanju-gun, Jeollabuk-do 55365, Republic of Korea; b National Institute of Fisheries Science(NIFS), 405, Gangbyeon-ro, Gunsan-si, Jeonbuk-do 54042, Republic of Korea; c Jeju National University, 102 Jejudaehak-ro, Jeju-si, Jeju Special Self-Governing Province 63243, Republic of Korea

**Keywords:** *Psychrobacter*, Genome, Vitamin B12 synthesis, Bioremediation, CRISPR-Cas, Probiotics, Antibiotic resistance, Cold-chain storage

## Abstract

This dataset provides the complete genome sequence of *Psychrobacter* sp. KFRI-CH2-11, isolated from Korean fermented anchovy, Myeolchi-jeotgal. Genomic analysis identified genes involved in Vitamin B12 biosynthesis, carbohydrate metabolism, CRISPR-Cas defense systems, and antioxidant activity, underscoring the strain's potential for use in food biotechnology. Additional genes linked to antibiotic resistance and bioremediation suggest adaptability in diverse environments, particularly cold-chain storage in the dairy and meat industry. PathogenFinder analysis confirmed the absence of pathogenicity-associated genes, validating the strain's suitability as a probiotic and biofortifying agent in food products.

Specifications Table

**Table:** 

Subject	Genomics
Specific subject area	Complete Genome sequence and genome Annotation of *Psychrobacter* for Probiotics, Biofortification, and Cold-tolerant microbes
Type of data	Tables, Figures
Data collection	High-quality genomic DNA was extracted using the Qiagen MagAttract HMW DNA Kit. Sequencing was conducted using Illumina HiSeq for short reads and PacBio for long reads, with assemblies performed using SPAdes and Canu for optimal genome completeness. Functional gene annotation was completed using KEGG, Prokka, and EggNog. PathogenFinder analysis confirmed the strain's safety for food biotechnology applications. Phylogenetic analysis was performed using MEGA10. the Genome-to-Genome Distance Calculator (GGDC) version 2.1 (http://ggdc.dsmz.de/distcalc2.php). Analysed using an ANI calculator tool (www.ezbiocloud.net/tools/ani) and AAI calculator tool (http://enve-omics.ce.gatech.edu/aai/).
Data source location	Psychrobacter sp. KFRI-CH2-11 was isolated from traditional Korean fermented anchovy (Myeolchi-jeotgal) at a local market on Chuja Island, Jeju-si, South Korea (33.9428757° N, 126.3213467° E).
Data accessibility	Repository name: NCBI (National Center for Biotechnology Information) GenBank Nucleotide database Data identification number: GenBank accession numbers CP151283.2 to CP151287.2 and BioProject accession number PRJNA861907 Direct URL to data: https://www.ncbi.nlm.nih.gov/nuccore/2720816003 https://www.ncbi.nlm.nih.gov/bioproject/PRJNA1097836
Related research article	None

## 1 Value of the Data

• The genome sequence of *Psychrobacter* sp. *KFRI-CH2-11* provides valuable insights into its biofortification and probiotic properties, relevant to the food industry.
• Genes associated with Vitamin B12 synthesis, carbohydrate metabolism, and antioxidant functions suggest the strain's potential to enhance nutritional content and stability in food products, particularly under cold-chain conditions.
• This data supports further research into the probiotic potential and metabolic versatility of psychrotolerant bacteria in food biotechnology, including dairy and meat applications.
• The genomic data offer foundational information for exploring industrial applications of *Psychrobacter* spp. in sustainable food production and microbial safety.
• Confirms safety through the absence of pathogenic genes, indicating suitability for human-consumable products

## 2 Background

The primary motivation behind compiling this dataset was to understand the unique genomic attributes of *Psychrobacter* sp. KFRI-CH2-11, a psychrotolerant bacterium isolated from traditional Korean fermented anchovy (Myeolchi-jeotgal). This study is grounded in psychrophilic microbial genomics, exploring organisms that adapt to cold environments and the potential applications of their metabolic pathways in food biotechnology [1]. *Psychrobacter* species are known for their resilience in low-temperature habitats, and KFRI-CH2-11 was analyzed specifically for its potential roles in biofortification, probiotic functionality, and environmental adaptability [2]. Genomic sequencing and functional annotation were conducted to map genes associated with vitamin B12 [3] biosynthesis, carbohydrate metabolism, CRISPR-Cas immune systems [4], antioxidant production, and bioremediation. This dataset, therefore, provides a foundational resource to further explore the strain's role in enhancing nutritional profiles in food applications and in contributing to food safety within cold storage contexts [5,6]. This data article complements key studies by providing a detailed genomic dataset suitable for comparative analyses of psychrotolerant bacteria and their industrial applications, offering broader insights into genetic adaptations that enable resilience in diverse environments [[7], [8], [9], [10]].

## 3 Data Description

**Fig. 1:**
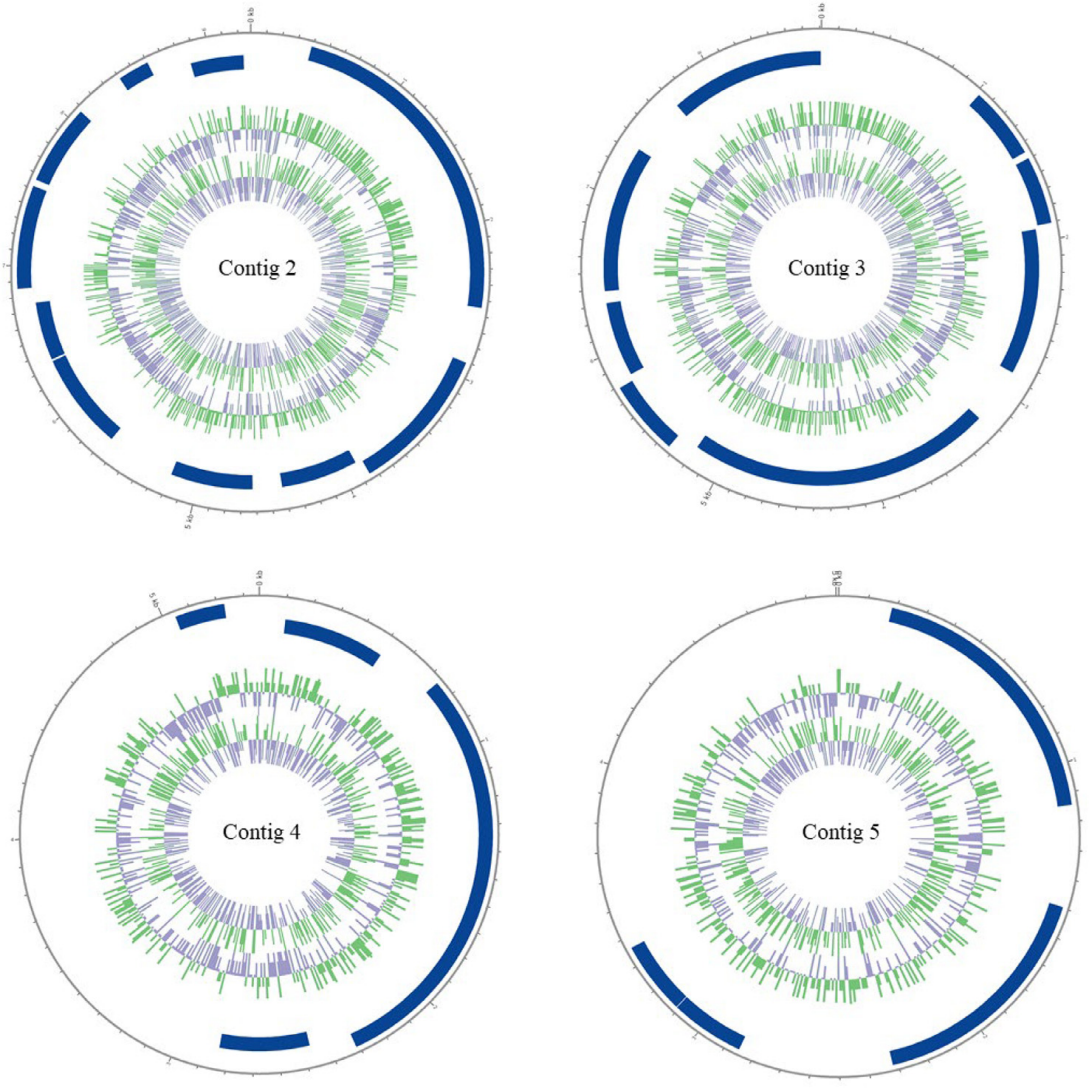
Four additional contigs no.2 to 5 Circular genome map of *Psychrobacter* sp. KFRI-CH2-11. The outermost ring represents the genome size in Mb. Circular map was drawn by applying contigs 2, 3, 4 and 5 annotation result. Marked characteristics are shown from outside to the center; CDS on forward strand (blue bars), CDS on reverse strand (blue bars), tRNA (green lines), rRNA (purple lines), GC content (green histogram) and GC skew (purple histogram).

**Fig. 2:**
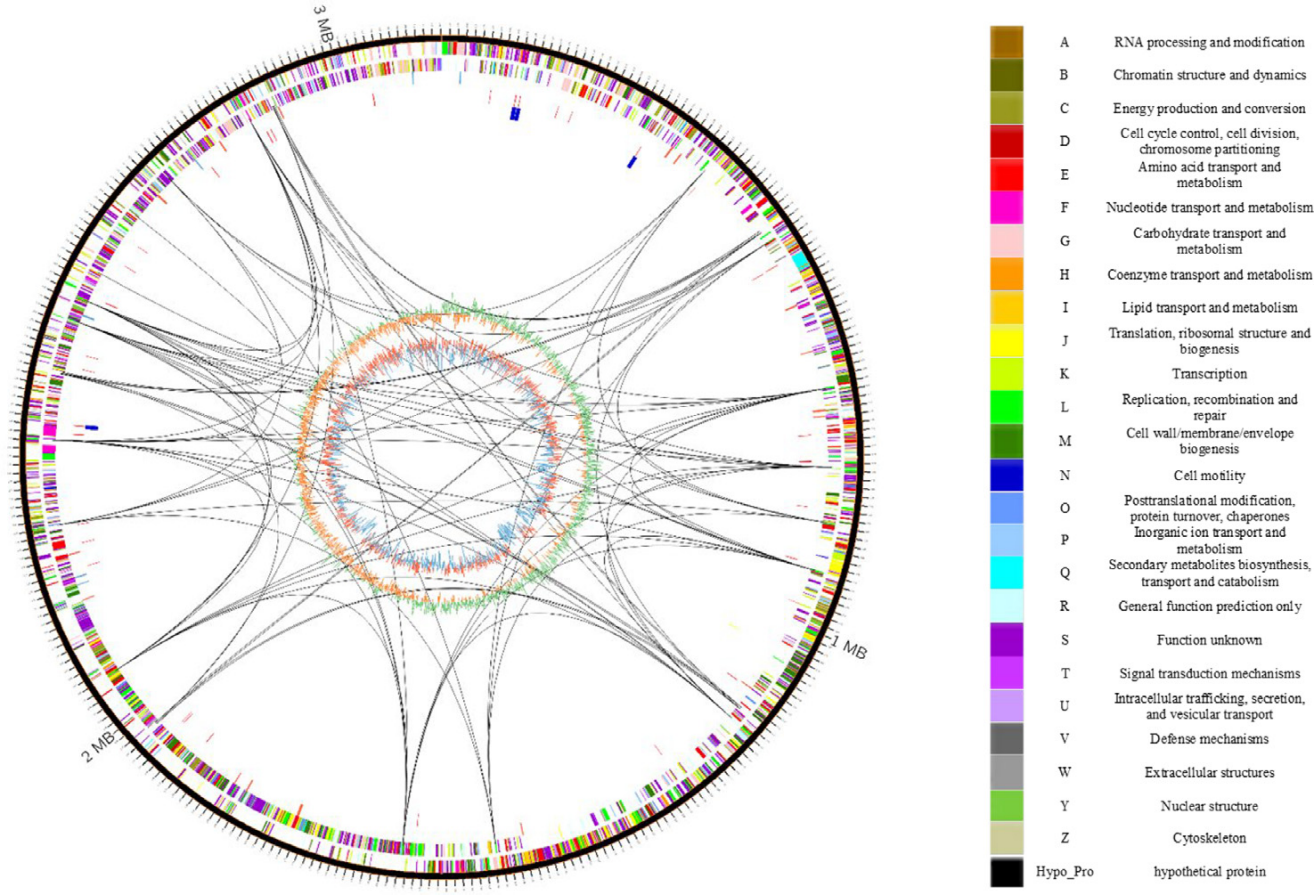
Circos plot of the Psychrobacter sp. KFRI-CH2-11 genome. The outermost rings display coding sequences (CDS) in both the forward and reverse strands, color-coded according to EggNOG functional categories (A-Z). Highlighted target genes include amylase (green), lipase (blue), and protease (red). The positions of tRNA, rRNA, and tmRNA are indicated by distinct colors: tRNA (red), rRNA (blue), and tmRNA (green). The inner rings represent GC skew (green and orange), illustrating guanine-cytosine asymmetry, and GC content (blue), with regions exceeding 50 % GC content marked in red. Internal connecting lines depict relationships between loci corresponding to the same gene site, indicating potential genomic interactions or colocalizations.

The genome of *Psychrobacter* sp. KFRI-CH2-11 comprised five contigs, including one chromosome and four additional contigs (Table 1) (Figs. 1, 2). The full genome sequence of chromosome spans 3131504bp with a GC content of 47.17 %, encoding 2630 genes, including 2565 protein-coding sequences, 49 tRNAs, and 12 rRNAs. The circular genome map reveals key genomic features, such as genes for vitamin B12 biosynthesis, carbohydrate metabolism, and the CRISPR-Cas system (Table 2). Functional annotation identified genes with potential industrial relevance, including *btuB, btuD,* and *cobO* for vitamin B12 biosynthesis, supporting nutritional biofortification. Carbohydrate metabolism genes (*lldD* and *mleN*) enable lactose utilization, relevant for dairy fermentation and probiotic applications. A CRISPR-Cas system composed of *cas1, cas2, cas3, cas6,* and *cse1/cse2* interference proteins provides immunity against foreign DNA, enhancing genomic stability. Additionally, genes for multidrug efflux transporters and penicillin-binding proteins confer antimicrobial resistance, supporting environmental resilience.

**Table 1:** Genome features of *Psychrobacter* sp. KFRI-CH2-11.

Feature	Value
Genome Length (bp)	3131,504
GC content (%)	47.17
Number of contigs	5⁎
tRNA	49
rRNA (5S, 16S, 23S)	4, 4, 4
ncRNA	4
Total genes (total)	2630
CDS (total)	2565
Number of CRISPR Arrays	3
N50 value	3,131,504
Coverage (%)	100
Genome Coverage (×)	249.5
Q30 value (%)⁎⁎	97.15
Human of Pathogenic proteins	ND⁎⁎⁎
Accession Number	CP151283.2 to CP151287.2

⁎ Number of contigs including one chromosome DNA and four contigs.

⁎⁎ Filtered dataset.

⁎⁎⁎ Not Detected.

**Table 2:** Genes related to identified in the genome of *Psychrobacter* sp. KFRI-CH2-11.

Gene Function	Gene Name	Locus Tag
Vitamin B12 Biosynthesis	*btuB*	AADH33_07010
	*btuD*	AADH33_07015
	*cobO*	AADH33_07020
Carbohydrate Metabolism	*lldD*	AADH33_01990
	*mleN*	AADH33_07665
Antioxidant Activity	superoxide dismutase (SOD)	AADH33_01380
	catalase	AADH33_02565
	catalase	AADH33_09785
CRISPR-Cas Defense System	*cas1*	AADH33_07880
	*cas2*	AADH33_07875
	*cas3*	AADH33_07910
	*cas6*	AADH33_07885
Bioremediation	*clpP*	AADH33_01365
	*lon_1*	AADH33_03060
	*mhpF*	AADH33_06630
Antibiotic Resistance	Multidrug resistance efflux	AADH33_08970
	*TetR/AcrR* family regulator	AADH33_05790
	Multidrug efflux MFS transporter	AADH33_05795
	*mrdA*	AADH33_03655
	*mrcB*	AADH33_04230

The Circos plot analysis of *Psychrobacter* sp. KFRI-CH2-11 displays key genomic features (Fig. 2). The outer rings represent coding sequences (CDS) in forward and reverse orientations, colored according to EggNOG functional categories. Specific target genes are highlighted, including amylase (green), lipase (blue), and protease (red). Positions of tRNA, rRNA, and tmRNA are marked, and the inner rings show GC skew (green and orange) and GC content (blue, with regions above 50 % in red). The genome contains a CRISPR-Cas system, including cas1, cas2, and cas3. Antibiotic resistance genes, such as multidrug efflux transporters and penicillin-binding proteins, are also present. Internal connections show links between loci of the same gene site.

**Fig. 3:**
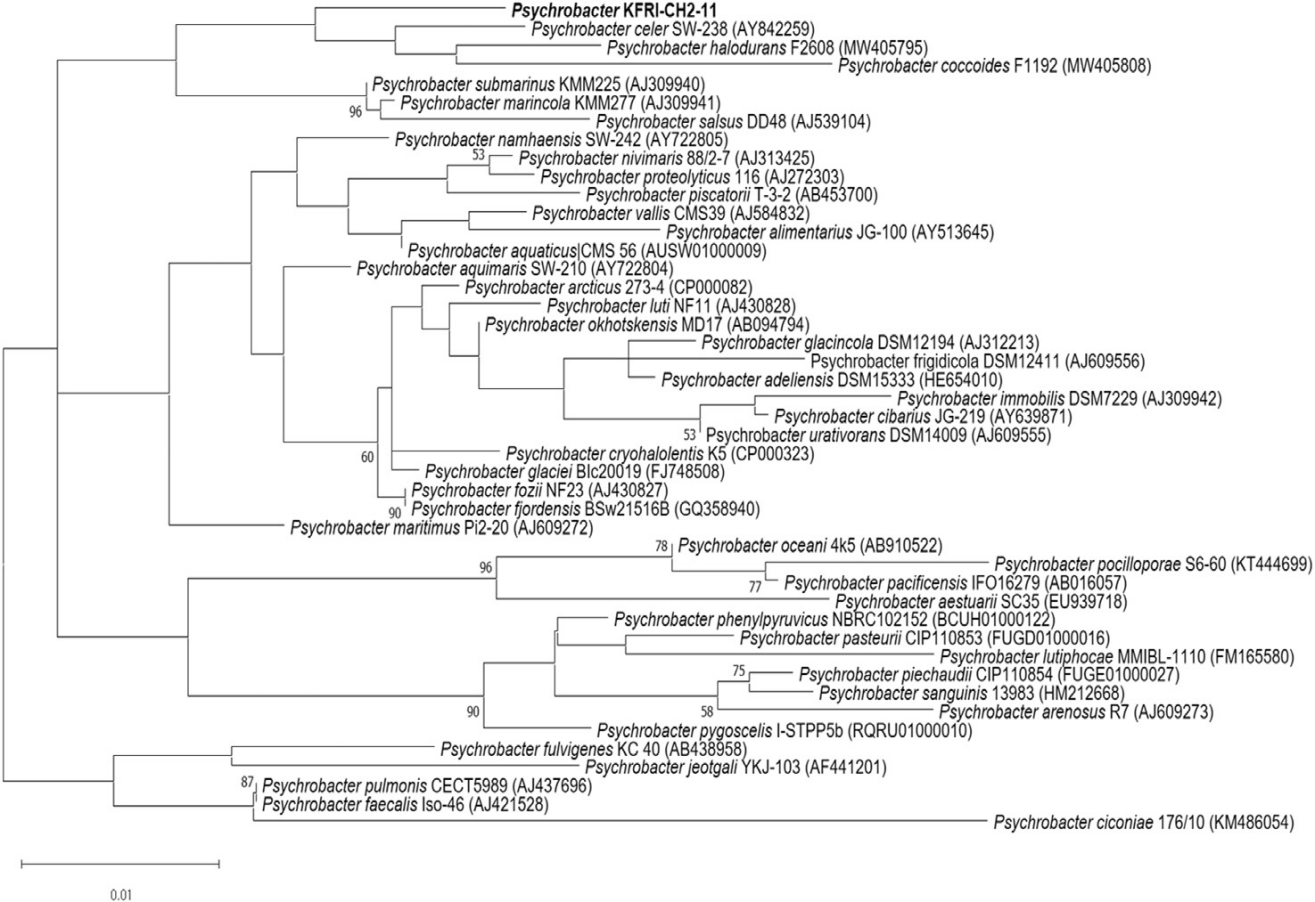
Phylogenetic tree constructed using the Maximum Likelihood method based on 16S rRNA gene sequences. The analysis includes Psychrobacter sp. KFRI-CH2–11 (BOLD) and closely related strains, with 1000 bootstrap replicates. The database used for the analysis is EzBioCloud 16S database (https://www.ezbiocloud.net/) version 20,230,823, and the Psychrobacter sp. sequences included in the bootstrapping process total 45. The bar represents 0.01 substitutions per nucleotide position, and bootstrap values greater than 50 % are shown at the branches.

*Psychrobacter* sp. KFRI-CH2-11 was identified by comparing 16S rRNA gene sequence with the closely associated publicly genus *Psychrobacter*. The highest 16S rRNA sequence similarity, as determined by the EzBioCloud database, occurred with *Psychrobacter celer* SW-238^T^ at 98.02 %, followed by *Psychrobacter halodurans* F2608 ^T^ at 97.67 % and *Psychrobacter coccoides* F1192^T^ at 96.99 %. Phylogenetic analysis using the maximum-likelihood method based on 16S rRNA gene sequences indicated that strain KFRI-CH2-11 fell within the genus *Psychrobacter* (Fig. 3). In addition, ANI, AAI and dDDH values were calculated for *Psychrobacter* sp. KFRI-CH2-11 and with closest associated strains (*Psychrobacter celer* SW-238^T^, *Psychrobacter halodurans* F2608 ^T^, *Psychrobacter coccoides* F1192^T^) and it was 77.52 - 97.22 % of the ANI values, 81.85 – 97.99 % of AAI values, and 25 - 75.8 % of dDDH values respectively.

## 4 Experimental Design, Materials and Methods

The study began with the collection of a traditional Korean fermented anchovy product, Myeolchi-jeotgal from at a local market on Chuja Island, Jeju-si, South Korea (33.9428757° N, 126.3213467° E). To isolate *Psychrobacte*r sp. KFRI-CH2-11, the sample was serially diluted and spread onto MA (marine agar) plates. The plates were incubated at 30 °C for 48 h, after which colonies with distinct morphological features were selected. These colonies were further purified by repeated streaking to ensure a pure culture of the target strain. Genomic DNA was extracted from the purified *Psychrobacter* sp. KFRI-CH2-11 was extracted using the Qiagen MagAttract HMW DNA Kit (Qiagen, Germany) following the manufacturer's protocol, followed by DNA quality assessment using spectrophotometric analysis. High-quality genomic DNA was then used for sequencing on both the Illumina HiSeq and PacBio platforms, providing a combination of short and long reads. Illumina data were quality-checked using FastQC (http://www.bioinformatics.babraham.ac.uk/projects/fastqc), and low-quality reads were trimmed with Trimmomatic v.0.39 [11]. Paired-end reads from Illumina sequencing were de novo assembled using SPAdes (v.3.13.0) [12], while PacBio long reads were assembled with the Canu v. 1.7 assembler to improve genome continuity and completeness. Quality control involved generating high-accuracy PacBio HiFi reads (≥99 % accuracy) by trimming adapters and merging reads from the same ZMW (Zero-Mode Waveguide), while Illumina data underwent adapter trimming and error reduction through the use of reversible terminator-bound dNTPs during sequencing. The genome size of *Psychrobacter* sp. KFRI-CH2-11 was estimated using k-mer analysis performed with Jellyfish v1.1.12, and the results were analyzed with GenomeScope employing the 4-peak model. The final genome assembly was assessed for accuracy and completeness, resulting in a highly reliable assembly with 99.4 % completeness. Open reading frames (ORFs) were predicted using Prodigal [13], a tool known for its accuracy in bacterial gene prediction. Functional annotation was performed using KEGG and EggNOG-mapper, which provided comprehensive orthologous group classification and functional categorization [14,15]. This analysis specifically focused on genes related to Vitamin B12 synthesis, the CRISPR-Cas immune system, carbohydrate metabolism, and bioremediation pathways, given the strain's potential applications in these areas. The pathogenic potential of *Psychrobacter* sp. KFRI-CH2-11 was assessed using PathogenFinder v1.1 (http://cge.cbs.dtu.dk/services/PathogenFinder/), a web-based tool designed to predict bacterial pathogenicity based on genomic data [5].

The Circos plot was generated to visualize the genomic structure and functional gene categories of *Psychrobacter* sp. KFRI-CH2-11. The plot was created using Circos software (version 0.69.8) on an Ubuntu 22.04 system [16]. Genomic data, including gene annotations, coding sequences (CDS), tRNA, rRNA, and other genomic features, were compiled from the genome assembly data. Functional gene categories were assigned based on the EggNOG database, categorizing each gene according to its predicted function. The Circos configuration file was meticulously prepared to incorporate all data layers, ensuring that each feature's position and color scheme matched the desired genomic layout. The plot was generated by executing the Circos command with the prepared configuration file, producing a high-resolution image. Finally, the Circos image was annotated with descriptions and a color-coded table summarizing the gene categories and target gene information, allowing for clear interpretation of each feature. The digital DNA-DNA hybridization (dDDH) value of *Psychrobacter* sp. KFRI-CH2-11 was determined using the Genome-to-Genome Distance Calculator (GGDC) version 2.1 (http://ggdc.dsmz.de/distcalc2.php) [17]. Average nucleotide identity (ANI) and average amino acid identity (AAI) from *Psychrobacter* sp. KFRI-CH2-11, *P. celer* SW-238, *P. coccoid*es F1192 and *P. halodurans* F2608 were analysed using an ANI calculator tool (www.ezbiocloud.net/tools/ani) and AAI calculator tool (http://enve-omics.ce.gatech.edu/aai/) [18. The 16S rRNA sequences were compared with related taxa obtained from the GenBank database and the EzBioCloud 16S database (https://www.ezbiocloud.net/). The resulting phylogenetic tree was constructed using maximum likelihood algorithms implemented in the MEGA10 software[19]. The Kimura 2-parameter model was employed to calculate phylogenetic distances, and bootstrap analysis was conducted using 1000 resampled datasets.

## Limitations

None.

## Ethics Statement

The authors have read and followed the ethical requirements for publication in Data in Brief and confirm that the current work does not involve human subjects, animal experiments, or any data collected from social media platforms.

## CRediT authorship contribution statement

**Myunglip Lee:** Conceptualization, Methodology, Writing – original draft. **Yucheol Kim:** Data curation, Software, Visualization. **Hae-Won Lee:** Software, Data curation. **Yukyoung Park:** Visualization. **Sunghun Yi:** Writing – review & editing.

## Data Availability

NCBIGenome Sequence (Original data). NCBISequence Info. (Original data).
